# Current perspectives of limbal‐derived stem cells and its application in ocular surface regeneration and limbal stem cell transplantation

**DOI:** 10.1002/sctm.20-0408

**Published:** 2021-05-05

**Authors:** Vivek Singh, Anil Tiwari, Abhinav Reddy Kethiri, Virender Singh Sangwan

**Affiliations:** ^1^ Stem Cell Biology Laboratory Center for Ocular Regeneration, L V Prasad Eye Institute Hyderabad India; ^2^ Department of Ophthalmology University of Pittsburgh School of Medicine Pittsburgh Pennsylvania USA; ^3^ Department of Cornea and Uveitis Dr. Shroff's Charity Eye Hospital New Delhi India

**Keywords:** corneal stromal stem cells, cultivated limbal epithelial transplantation, limbal stem cell deficiency, limbus, simple limbal epithelial transplantation, stem cells

## Abstract

Limbal stem cells are involved in replenishing and maintaining the epithelium of the cornea. Damage to the limbus due to chemical/physical injury, infections, or genetic disorders leads to limbal stem cell deficiency (LSCD) with partial or total vision loss. Presently, LSCD is treated by transplanting limbal stem cells from the healthy eye of the recipient, living‐related, or cadaveric donors. This review discusses limbal‐derived stem cells, the importance of extracellular matrix in stem cell niche maintenance, the historical perspective of treating LSCD, including related advantages and limitations, and our experience of limbal stem cell transplantation over the decades.

## Graphical abstract

Advancement in the stem cell therapy for treating limbal stem cell deficiency (LSCD) helps in improved corneal vision. Apart from surgical procedure of transplanting the limbal cells or tissues, several types of cells are assessed for enhanced therapeutic approach. Other future possibilities like limbal niche reconstruction, development of artificial corneas and bioengineered extracellular matrix (ECM) would further enrich the stem cell treatment.

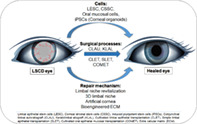

**Significance statement**

Limbal stem cells are the adult stem cells located in the basal epithelial layer of the corneal limbus that aid in the renewal of the corneal epithelium. Damage to limbal stem cells affects corneal epithelial regeneration and the subsequent invasion of conjunctival epithelium and neovascularization of the corneal surface. This causes a disease called limbal stem cell deficiency, which leads to blindness in the cornea. This article summarizes the types of stem cells present in the limbus and their cell culture techniques. Recent advancements in stem cell treatment for corneal pathologies are discussed, with historical outlook and clinical significance.

## 1 INTRODUCTION

Stem cells have the self‐renewal ability and potency to differentiate into specific cell types or an entire organism. Adult stem cells (ASCs) are populations of cells involved in an internal repair mechanism generating replacement for cells lost through the wound healing process, wear and tear, injury, and diseases.^1^ They reside at specific anatomical locations and may remain quiescent for long periods until activated by their need to maintain tissue homeostasis.^2^ Stem cells for the cornea are present at the corneoscleral limbus. Two population of ASCs exists in the limbal niche, epithelial and stromal stem cells, referred as limbal epithelial stem cells (LESCs)^3^ and corneal stromal stem cells (CSSCs),^4^ respectively. These stem cell populations are essential to maintain corneal transparency.^5^ Additionally, the limbal niche also harbors early transient amplifying cells, melanocytes, and Langerhans cell.^6^, ^7^, ^8^ As per the recent global consensus by the International Limbal Stem Cell Deficiency Working Group, they concluded that “Autologous limbal stem cell transplantations using the least amount of donor tissue, such as simple limbal epithelial transplantation (SLET), ex vivo‐cultivated autologous LSC, and modified conjunctival limbal autograft (CLAU), are preferred over other surgical treatments for unilateral or subtotal bilateral limbal stem cell deficiency (LSCD) or whenever feasible because of better long‐term outcomes and fewer complications”.^9^

This review summarizes the recent significant progress based on our experience and literature survey on ocular surface stem cells, their culture and expansion, corneal epithelial regeneration, limbal stromal cells, and the role of limbal niche. We have also discussed the associated challenges and need of various stem cells in ocular therapies.

## 2 CULTURE AND EXPANSION OF LIMBAL‐DERIVED STEM CELLS

Limbal stem cells are the well‐characterized cells of the ocular surface^10^ other than conjunctival progenitors whose anatomical location remains elusive.^11^ LESCs and CSSCs can be admirably identified in vitro with different morphological and slow‐cycling properties of the cells.^12^ Limbal epithelial cultures used for transplantation in treating ocular surface burns are known to contain stem cells that are identified by the positive expression of the p63α^13^ and ABCG2.^14^ The recent discovery of ABCB5 as a marker for limbal stem cells and its tendency to localize with p63α could aid in exclusive isolation of stem cell populations in limbus.^15^ The properties of LESC were further retained or enhanced by their culture on feeder layers that includes gamma‐irradiated 3T3 cells, human embryonic fibroblasts, or human amniotic epithelial cells. Interestingly, intrinsic feeder layers were also observed in limbal epithelial cultures that help in the maintenance of the stemness in the limbal explant culture system.^16^, ^17^

### 2.1 Limbal epithelial cells

In vitro cell expansion potential of the limbal tissues had revealed that tissue obtained from living subjects had a higher capability to initiate cell growth than the preserved cadaver tissues.^18^ This decrease in cell viability of the cadaver tissues could be attributed to preservation outside their nativity. Whereas better cell growth with fresh tissue than cadaver tissue is comprehensible, it seems that limbal stem cells remain viable in the limbal niche during storage of cadaver corneal tissue for weeks.^19^ We had also observed from our study that 60% of the cadaver tissues were able to initiate the cell growth in contrast to 90% of the live tissues. Interestingly, these cadaver tissues that have initiated cell growth compete with the live tissue in terms of cell expansion and epithelial cell integrity, demonstrating viable limbal stem cell populations despite preservation. Moreover, the epithelial cell sheets developed from the limbal explants ex vivo showed greater integrity, as evident from the rich development of desmosomes, hemidesmosomes, and E‐cadherins.^20^, ^21^

Furthermore, a study reported by Ekpo et al had cultured the cadaver tissues adjacent to the limbus, that is, toward corneal (L.cor) and conjunctival (L.conj) sides. Interestingly, they have noted that the cells cultured from the L.conj had more growth potential and stemness than L.cor, as evident from p63α expression.^22^ Though live limbal tissue has desirable cell growth, cadaver limbal rims obtained either during corneal keratoplasties or from stored eye banking tissues also serves as a viable alternative for transplantation purposes.

### 2.2 Limbal niche cells

**FIGURE 1:**
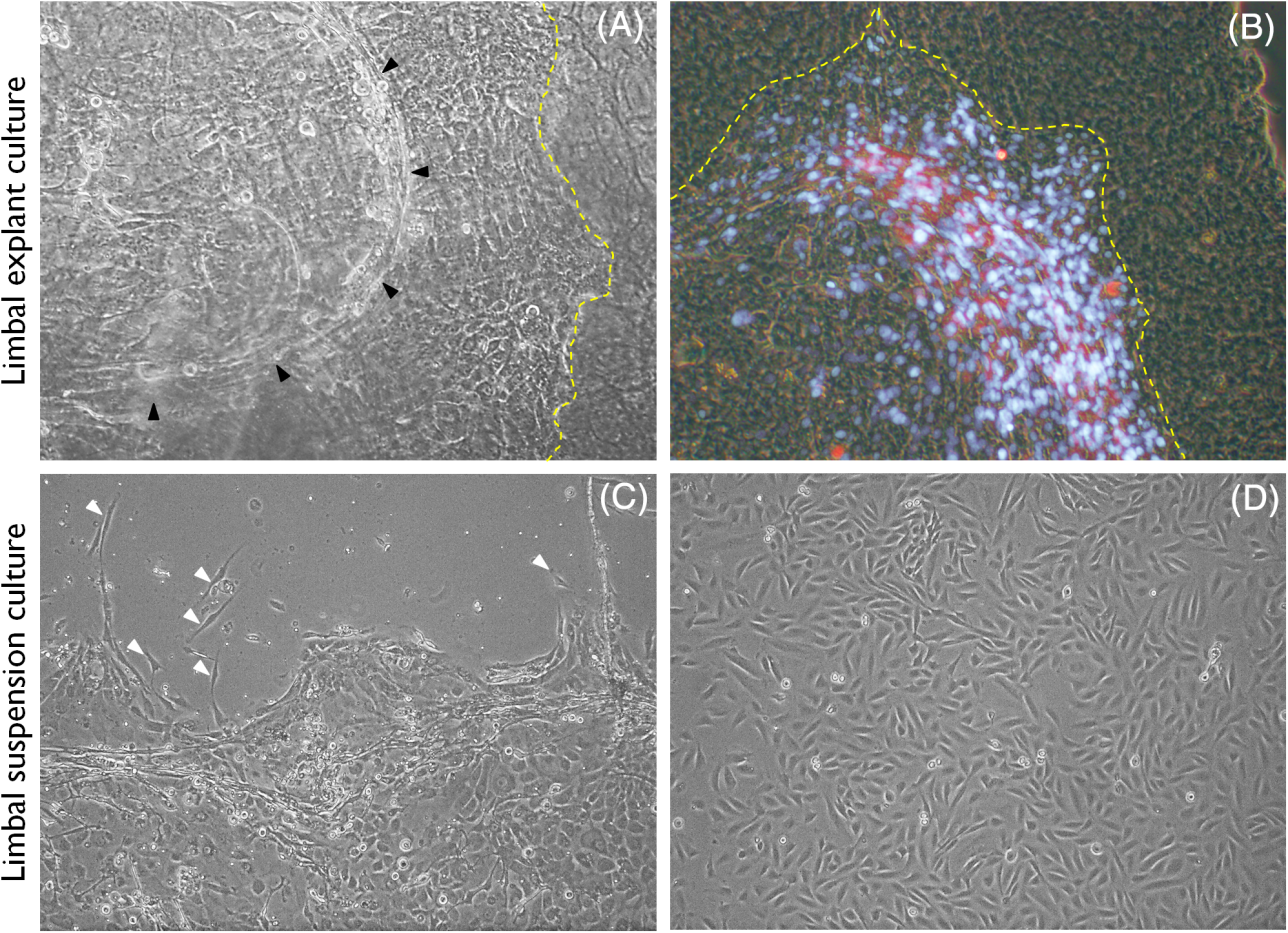
Ex vivo expansion of limbal epithelial cells and stromal cells. A, Human limbal tissue cultured on human amniotic membrane. At the edge of the epithelial cell expansion, stromal cells can be observed as a streak (black arrow heads) that always tends to move forward with the epithelial cells expand on the membrane. B, Some of the stromal cells in these streak can be seen positively expressing ABCG2 (Red stain; Blue indicates 4′,6‐diamidino‐2‐phenylindole [DAPI] staining). Yellow dotted line in (A) and (B) indicate the demarcation of the cells grown (left) and amniotic membrane (right). C, Human limbal tissue cultured after enzymatic digestion on tissue culture dish shows the epithelial cells. At day 8, stromal cells (white arrow heads) can be seen emerging from beneath the epithelium. D, Stromal cells devoid of epithelium at day 15 of the second passage

CSSC exhibits mesenchymal stem cell‐like properties,^23^ although they are of neurocrest origin and are identified as side population cells expressing ABCG2 and PAX6. While CSSCs are multipotent mesenchymal cells, LESCs are confined to deriving corneal/limbal epithelium.^4^ Though the primary culture of CSSC survives longer passages (40‐50), they may lose their plasticity with the increase in senescence and therefore are preferred at earlier passages in cell therapies.^24^ Our in vitro experiments of limbal cultures had shown that these cells are predominantly identified as a streak at the expanding edge of the expressing ABCG2 (Figure 1A,B). In enzymatically digested limbus tissue cultures, we observed that the stromal cells emerge from the epithelial sheet at late stages of culture, which are evidently visible in further passages (Figure 1C,D).

Ex vivo culture of the limbal tissue used for transplantation involves the use of irradiated murine fibroblasts as feeder layers, fibrin sealant, or the human amniotic membrane (AM) as a scaffold for cell growth. While feeder layers have the risk of xenogeneicity, AM carries the risk of disease transmission and batch variations. However, no adverse events have been reported in the use of different substrates.^25^ Recent studies have shown that fiber hydrogel and serum‐free media synergize to provide an optimal environment for keratocyte phenotype growth as well as the regeneration of damaged corneal stroma.^26^, ^27^ Interestingly, stromal cells cultured on two‐dimensional (2D) silk fibroin sheets and stacked to provide three‐dimensional (3D) structure leverages both 3D microenvironment and stromal cell sufficiency.^28^

## 3 EXTRACELLULAR MATRIX NICHE FOR ENHANCED CELL THERAPY

The extracellular matrix (ECM) harboring ASCs comprises adhesive proteins, proteoglycans, polysaccharides, and structural proteins: fibronectin, laminin, and tenascin‐c. The shape and composition of ECM depend upon the tissue, developmental stages, and pathological conditions, thereby affecting the overall fate of the stem cells. ECM orchestras the native 3D environment surrounding ASCs, and neighboring cells and also serves as an active reservoir of soluble factors, thereby modulating cellular behavior.^29^ Besides being a dynamic microenvironment, ECM provides biophysical properties essential for ASC maintenance. In the case of ocular surface wounds that may destroy the limbal niche, revitalization of the microenvironment along with limbal cell transplantation had shown promising results. It has been well established that stem cells thrive in 3D structures (cell spheres) and differentiate in two‐dimensional cultures. Therefore, 3D reconstruction of the limbal niches^30^ had gained attention in fabricating artificial corneas or scaffolds to support stem cell maintenance.^31^

Additionally, replacement of damaged ECM with AM or bioengineered ECM would provide enhanced cell therapies.^32^, ^33^ Therefore, it is essential to understand the ASC‐ECM interaction in maintaining niche, which provides a conducive milieu for in vitro expansion of ASCs.^34^, ^35^ Understanding the specific components of ECM, its role in regulating stem cell behavior in different tissues can be deciphered from the in vivo studies and with the use of engineered in vitro niche.^36^ Similarly, ECM is vital for the physiological renewal of the ocular surface/limbal microenvironment and plays a crucial role in the function and maintenance of the limbal niche.^37^ The limbal niche comprises well‐organized ECM, signaling drivers, and niche cells such as melanocytes, immune cells, vascular cells, nerve cells, and stromal cells, which are compromised in certain hereditary conditions or severe insults to the limbus.^32^, ^38^, ^39^, ^40^, ^41^ Bioengineered corneas are fabricated using polyethylene glycol to generate a niche like structure^42^ or entire corneal stroma with keratocytes embedded in them.^43^ Porous hydrogels similar to collagen structure were also developed by the Griffith's group that amalgamates with natural fibrin compound to form LiQD corneas.^44^ These studies will potentially open new avenues for creating more accurate in vivo niche features by applying in vitro system, enabling a better understanding of the biology and efficacy of stem cell‐based therapies.

## 4 HISTORICAL PERSPECTIVE AND PROGRESSIVE SHIFT IN LIMBAL STEM CELL TRANSPLANTATION

The concept of limbal stem cells in treating LSCD was clinically applied by Kenyon and Tseng, where the conjunctiva with limbus tissue was used for ocular surface reconstruction in patients with chemical injuries.^45^ Since then, chemical burns of the eye had been challenging with complexity and variation among the patients. Much emphasis has been given to understand the extent of LSCD firsthand before deciding on the therapeutic approach. Clinical presentation of ocular surface chemical burns has been graded using either Roper‐Hall's or Dua's classification to understand the extent of limbal tissue damage.^46^ With further clinical advancements, in vivo confocal microscopy identifies specific cell types for accurate diagnosis of LSCD.^47^ Apart from the severity of chemical burns, other factors such as lid deformities, tear film abnormalities, and inflammation control have been key factors in managing LSCD.^48^

Therapeutic options for LSCD include CLAU that involves healthy conjunctival and limbal tissue patches obtained by sectoral excision from the donor and transplanted in to the affected eye.^45^ Kerato‐limbal allograft mimicks CLAU except for the donor tissue source used, usually a cadaver tissue or living‐related donor^49^ and requires immunosuppression. Amniotic membrane grafting (AMG) is a simpler technique used for acute cases of chemical burn that reduces inflammation and promotes healing and scar‐free regeneration of the epithelium. AMG could also be applied in conjunction with other therapeutic cells for better efficacy.^50^ Currently, cell‐based therapies such as cultivated limbal epithelial transplantation (CLET) and SLET are the preferred choice for better clinical outcomes and less donor complications.

With the drawbacks of obtaining larger grafts and risking the donor eye in CLAU, Pellegrini et al had generated corneal epithelium by ex vivo serial cultivation of cells using a 1 to 2 mm^2^ autologous or cadaver limbal donor tissue on irradiated 3T3 cells. These cells were further transplanted on the patient's affected eye giving rise to the technique of CLET.^51^ The cell growth substrate was later modified to the AM,^52^ which avoids the xenogeneicity in transplantation. The advantage of CLET is the ex vivo increase of cell population that could be assessed for cell phenotypes and the percentage of stem cell pool ascertaining the successful outcomes of CLET.^13^ Besides, any gene related defects could be corrected and then transplanted in CLET.^53^ The largest case series of CLET had reported success rates of 71% (142 of 200 eyes),^54^ 76% (443 of 583 eyes),^55^ or 70% (815 of 1164 eyes)^56^ with key successful outcomes as the transparent and avascular cornea. In cases where CLET fails, a repetitive surgery of the same had demonstrated a success rate of 66% (33 of 50 eyes) with improvement in vision by 76%.^57^ Additionally, the long‐term follow‐up of CLET in two individual studies reported a survival rate of 76.6%^13^ and 71%,^54^ 10 years after the surgery, indicating the cell sustainability and maintenance of corneal homeostasis. Overall, CLET is a proven successful technique to treat LSCD with a shortened time of corneal re‐epithelialization, repeatability of the procedure, and rare donor complications. Therefore, commercial marketing of this technique as “Holoclar” has been approved by the European medical agency for treating LSCD. The efficacy of the Holoclar treatment lies in reliably identifying the holoclones that highly express ∆Np63α and its significant association with clinical success.^58^ However, the costs involved in CLET that require cell culturing facility and time needed to culture for each patient are the receding factors for public outreach.^59^ Recently, limbal stem cells from cadaver tissues have been isolated exclusively based on the positive expression of ABCB5, expanded in vitro as advanced therapy medicinal product (ATMP), and transplanted in clinical trials in patients with LSCD.^60^

Autologous limbal cultures were the first stem cell‐based therapy approved by ATMPs (in 2015), and it has also received marketing authorization as Holoclar from the European Medicine Agency. Holoclar uses the patient's own (autologous) limbal stem cells to treat unilateral LSCD.^58^ Studies by Luca and Pellegrini showed that the clinical success of Holoclar treatment had a positive correlation with the percentage of holoclones present in the limbal culture.^13^, ^61^ Many other groups worldwide have used limbal cultures to treat ocular burns, often with different culture systems.^1^

**FIGURE 2:**
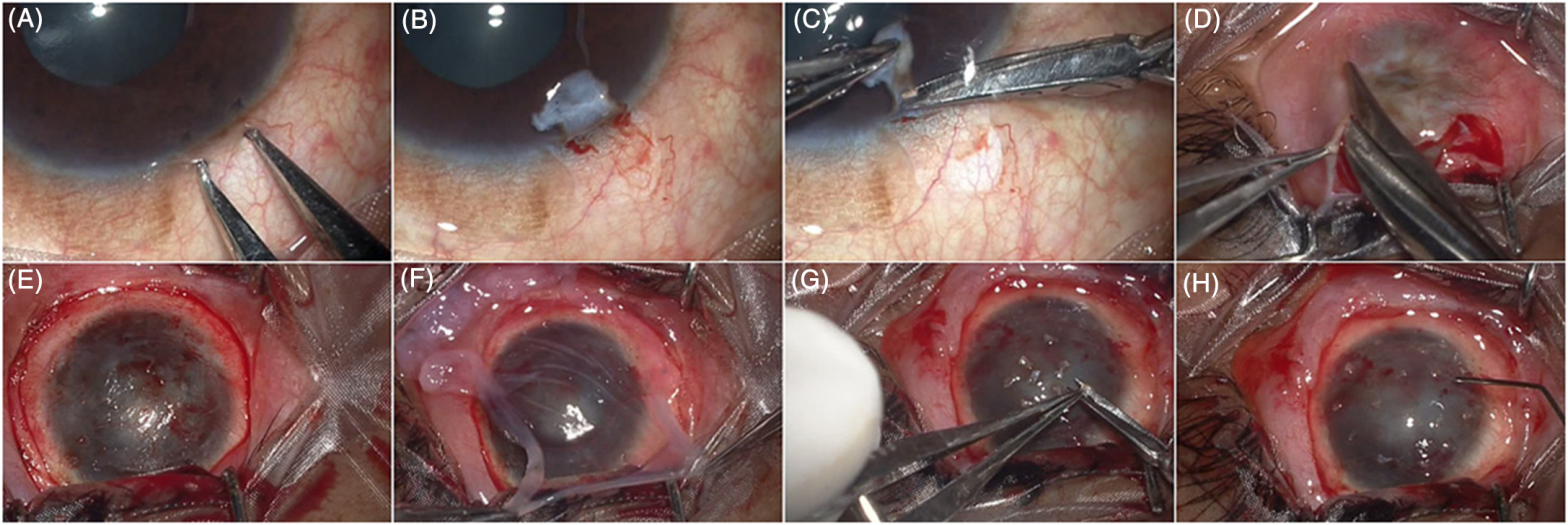
Clinical photographs showing the surgical technique of limbal biopsy from the donor eye and transplantation of the limbal tissue on the recipient eye. A, A 2 × 2 mm area is marked across the superior limbus of the donor eye. B, A subconjunctival dissection is carried out 1 mm into the clear cornea. C, The limbal tissue is excised. D,E, A peritomy is performed and the fibrovascular pannus is excised from the recipient ocular surface. F, A human amniotic membrane graft is placed on the bare ocular surface and secured to it with fibrin glue. G,H, The donor limbal tissue is cut into 8 to 10 small pieces and secured to the amniotic membrane overlying the cornea with fibrin glue. Reprint from Sangwan et al.^62^

With the knowledge and experience from CLET, our group had developed SLET, a novel method of expanding the limbal cells in vivo directly on the patient's affected eye while continuing the use of AM as substrate. This technique involves spreading AM on the ocular surface after removing the fibrovascular pannus and placing small pieces of donor limbal tissues uniformly over the corneal surface (Figure 2). Cell outgrowth from the limbal explants on the ocular surface after SLET merges into each other similar to in vitro cell expansion and corneal re‐epithelialization typically happens in 2 weeks. Initial surgery with six patients had demonstrated 100% success at 6 months follow‐up.^62^ This strategy is similar to epidermal skin grafting where small skin tissue pieces spread across the larger wounded areas promotes skin pigmentation/re‐epithelialization. A large, single‐center case series of at least 1 year follow‐up has shown a 76% (95 of 125 eyes) success rate with 75% improvement in the patients' visual acuity and a median follow‐up of 1.5 years.^63^ Another multicenter analysis had shown an 84% (57 of 68 eyes) success rate at 1 year follow‐up.^64^ SLET, when compared to CLAU, had similar success rates and ocular surface parameters postsurgery.^65^ Failure of SLET had most likely appeared in the first 6 months after the surgery. Loss of the transplanted grafts is also associated with the recurrence of conjunctivalization and failure of SLET.^66^ In our experience, risk factors of transplantation failure include preoperative conditions of the patient such as dryness of the eye, unaddressed pre‐existing conditions such as symblepharon involvement or lid deformities, persistent epithelial defects, and simultaneous penetrating keratoplasty.^63^, ^67^, ^68^ AM also stands as a critical factor in the success of SLET since the limbal explant growth and survival is dependent on the AM.^69^

Ex vivo cultivation of the corneal epithelial cells from limbal tissue requires a cell culture laboratory, and therefore CLET is an expensive procedure with less patient outreach due to lack of facilities in the hospital setting and ethical regulations. SLET, on the other hand, does not need a cell culture laboratory and is also a single‐stage surgery making it inexpensive to CLET. Importantly, SLET has comparable or even better results compared to CLAU and CLET, in addition to the ease of ophthalmologists to perform this surgical technique.^67^, ^70^ Additionally, SLET is being used to manage ocular surface disorders such as pterygium^71^ or ocular surface squamous neoplasia^72^ that involves damage to the limbus. Limitations of SLET are in cases of persistent epithelial defects observed even after multiple surgeries, and therefore, CLET can be considered in such cases to provide adequate epithelial cells. Additionally, SLET is usually avoided or is combined with CLAU in reconstruction of a complex ocular surface or severe symblepharon. In cases of bilateral LSCD, allogeneic CLET/SLET are available, which carriers a greater risk of rejection and eventual requirement of immunosuppression. Cultivated oral epithelial mucosal transplantation (COMET) serves as a better redressal to treat bilateral LSCD with no immune suppression requirement. Nevertheless, persistent epithelial defects and graft failure were higher in COMET compared to allo‐CLET.^73^

With the advent of CLET and SLET, several other cell sources are also being explored in treating LSCD. Specifically, induced pluripotent stem cells (iPSCs) offer a promising approach to generate mature corneal/limbal epithelium with 3D corneal organoids.^74^ Once standardized, iPSC‐derived corneal/limbal epithelial cells could be an unlimited source for limbal cell therapies. However, all these techniques have their advantages and limitations (Table 1) and are used depending on the condition of the patient. Furthermore, noncellular therapies such as patient‐derived hemo or AM derivatives and soluble growth factors are the emerging approaches in the regeneration of the ocular surface in circumventing allogeneic complications.^82^ Another impending, potent source of stem cells for treating LSCD is the limbal stromal‐derived cells. Though these cells were not characterized during CLET, they were concealed constituent of the cultures and could have played a role in wound healing after transplantation. Gene expression profiles of the isolated limbal cultures derived stromal cells had similar expression profiles to bone marrow‐derived mesenchymal cells.^23^ These stromal cells isolated from peripheral cornea or limbus also showed keratocyte markers' expression and could differentiate to chondrogenic or adipogenic cells,^12^, ^24^ similar to mesenchymal cells. Basu et al had derived the human limbal biopsy stromal cells and transplanted them on the wounded mice cornea with successful prevention of scars indicating the role of stromal fibroblasts in rearranging the stromal ECM. Moreover, these cells were also capable of reversing the already existing scars into a clear cornea, as evident in nitrogen injured scar models of mice.^83^ Interestingly, the collagen reorganization of the wounded mice cornea and the lamellar structure was indistinguishable from the normal cornea making the limbal stromal cells a potent therapeutic for treating corneal stromal blindness.^84^ The immunomodulation ability of limbal stromal‐derived cells makes them an excellent source for treating corneal scars and acute chemical injuries of the cornea.

**TABLE 1:** Advantages and limitations of surgical techniques to treat limbal stem cell deficiency

Technique	Source of tissue	Benefits	Limitations	Reference
CLAU	Conjunctiva and limbus	Larger patch of conjunctiva can be used for ocular surface reconstruction	Larger limbal grafts, risk at donor site complications	75
AMG	Amniotic membrane obtained during cesarean	Anti‐inflammatory properties, no immune rejection, natural biological patch	Storage and risk of disease transmission	76
CLET	Limbal biopsy	Smaller donor limbal tissue. Characterization and increasing the number of epithelial or stem cells. Epithelial cell sufficiency in recurrent epithelial defects, Gene editing	Cell culture expert dependency. Cost involved in maintenance of CGMP facility and hence increase in surgical costs.	13
SLET	Limbal biopsy	No requirement of a CGMP facility. Single stage procedure. Smaller donor limbal tissue.	Risk of loss of donor limbal tissue. Not suitable for persistent epithelial defects or ocular surface reconstruction.	62, 67
COMET	Oral buccal mucosal epithelium	Treatment of bilateral LSCD. Can avoid allogeneic immunosuppression	Poor differentiation to corneal epithelial cell type. Risk of dry‐eye conditions.	77, 78
Keratoprosthesis	Biological (dental or osteo), Biocompatible (polymethyl methacrylate) with cadaver cornea	Treatment of bilateral LSCD, Immediate vision	Frequent conjunctivalization, risk of glaucoma	79
iPSC	Skin punch biopsy	Ability to generate mature corneal epithelium or organoids. Autologous tissue can be used in bilateral LSCD.	Risk of tumorigenic potential. Requirement of robust cell characterization.	74
Mesenchymal stem cells	Bone marrow	Sufficiency of autologous tissue for transplantation. Anti‐inflammatory properties.	Cannot differentiate to corneal epithelial cells	80, 81

Abbreviations: AMG, amniotic membrane grafting; CGMP, Current Good Manufacturing Practice; CLAU, conjunctival limbal autograft; CLET, cultivated limbal epithelial transplantation; COMET, cultivated oral epithelial mucosal transplantation; iPSC, induced pluripotent stem cell; LSCD, limbal stem cell deficiency; SLET, simple limbal epithelial transplantation.

## 5 CONCLUSION

The biggest challenge for the stem cell culture‐based therapies lies in the availability of eye bank tissue, AM, maintenance, transportation, and costs involved in the stem cell culture. Besides, maintenance of cells in the long‐term storage and expansion of these cells for clinical use may alter their stem cell properties. After all, the current regulatory hurdle and Good Manufacturing Practice (GMP) compliance take a long time, and the costs involved are enormous; therefore, SLET (for LSCD) still seems very promising and cost‐effective. For many other incurable corneal pathologies, CSSCs or the bone marrow‐derived stem cells would help overcome the limitations of limbal transplantation. The need of the hour is reliable, stable, and over the shelf treatment for corneal perforation, haze, and inflammation, including ocular stem cell deficiency like the teardrop bottle.

## CONFLICT OF INTEREST

The authors declared no potential conflicts of interest.

## AUTHOR CONTRIBUTIONS

V.S.: conception and design, financial support, administrative support, collection and/or assembly of data, provision of study material or patients, collection and/or assembly of data, data analysis and interpretation, manuscript writing, final approval of manuscript. A.T., A.R.K.: collection and/or assembly of data, data analysis and interpretation, manuscript writing. V.S.S.: conception and design, financial support, administrative support, provision of study material or patients, data analysis and interpretation, manuscript writing, final approval of manuscript.

## Data Availability

Data sharing is not applicable to this article as no new data were created or analyzed in this study.
